# Supplementary material for: Heparin-based hydrogel scaffolding alters the transcriptomic profile and increases the chemoresistance of MDA-MB-231 triple-negative breast cancer cells
Source: Biomater Sci. 2020 Feb 13;8(10):2786–96. doi: 10.1039/c9bm01481k (PMC7497406; doi:10.1039/c9bm01481k)
Supplement: Supplementary file 2 [file BM-008-C9BM01481K-s002.zip › Supplementary File 4/HP-BhydrogelvControl/Pathways/my_analysis.Gsea.1545201079907/HALLMARK_CHOLESTEROL_HOMEOSTASIS.html]

Details for gene set HALLMARK\_CHOLESTEROL\_HOMEOSTASIS[GSEA]

|  || Dataset | expr.class.cls#HYD\_versus\_CONTROL.class.cls#HYD\_versus\_CONTROL\_repos |
| Phenotype | class.cls#HYD\_versus\_CONTROL\_repos |
| Upregulated in class | CONTROL |
| GeneSet | HALLMARK\_CHOLESTEROL\_HOMEOSTASIS |
| Enrichment Score (ES) | -0.35789117 |
| Normalized Enrichment Score (NES) | -1.4363354 |
| Nominal p-value | 0.032490976 |
| FDR q-value | 0.029435188 |
| FWER p-Value | 0.19 |
Table: GSEA Results Summary

  

Fig 1: Enrichment plot: HALLMARK\_CHOLESTEROL\_HOMEOSTASIS      
 Profile of the Running ES Score & Positions of GeneSet Members on the Rank Ordered List

  

| PROBE | DESCRIPTION (from dataset) | GENE SYMBOL | GENE\_TITLE | RANK IN GENE LIST | RANK METRIC SCORE | RUNNING ES | CORE ENRICHMENT || 1 | FASN | na |  |  | 563 | 2.266 | 0.0165 | No |
| 2 | ABCA2 | na |  |  | 1340 | 1.660 | 0.0096 | No |
| 3 | ATXN2 | na |  |  | 1501 | 1.575 | 0.0332 | No |
| 4 | MVD | na |  |  | 1905 | 1.404 | 0.0406 | No |
| 5 | PCYT2 | na |  |  | 2176 | 1.286 | 0.0526 | No |
| 6 | ATF3 | na |  |  | 2583 | 1.150 | 0.0547 | No |
| 7 | CHKA | na |  |  | 3515 | 0.914 | 0.0246 | No |
| 8 | TNFRSF12A | na |  |  | 3645 | 0.883 | 0.0357 | No |
| 9 | SEMA3B | na |  |  | 3816 | 0.847 | 0.0440 | No |
| 10 | LSS | na |  |  | 4238 | 0.764 | 0.0375 | No |
| 11 | LDLR | na |  |  | 5135 | 0.608 | 0.0030 | No |
| 12 | ETHE1 | na |  |  | 5531 | 0.548 | -0.0065 | No |
| 13 | ATF5 | na |  |  | 5960 | 0.491 | -0.0189 | No |
| 14 | GUSB | na |  |  | 6634 | 0.403 | -0.0459 | No |
| 15 | EBP | na |  |  | 7038 | 0.355 | -0.0598 | No |
| 16 | SREBF2 | na |  |  | 7209 | 0.338 | -0.0618 | No |
| 17 | GNAI1 | na |  |  | 7970 | 0.251 | -0.0964 | No |
| 18 | CPEB2 | na |  |  | 8549 | 0.199 | -0.1226 | No |
| 19 | FBXO6 | na |  |  | 9028 | 0.150 | -0.1445 | No |
| 20 | FADS2 | na |  |  | 9328 | 0.112 | -0.1579 | No |
| 21 | TRIB3 | na |  |  | 9434 | 0.099 | -0.1614 | No |
| 22 | ECH1 | na |  |  | 9762 | 0.069 | -0.1771 | No |
| 23 | DHCR7 | na |  |  | 10032 | 0.036 | -0.1904 | No |
| 24 | ACSS2 | na |  |  | 10092 | 0.031 | -0.1928 | No |
| 25 | FDPS | na |  |  | 10354 | 0.012 | -0.2062 | No |
| 26 | ALCAM | na |  |  | 10706 | -0.030 | -0.2240 | No |
| 27 | STX5 | na |  |  | 11073 | -0.074 | -0.2416 | No |
| 28 | PLAUR | na |  |  | 11509 | -0.112 | -0.2621 | No |
| 29 | MVK | na |  |  | 11949 | -0.170 | -0.2816 | No |
| 30 | HMGCS1 | na |  |  | 12129 | -0.190 | -0.2871 | No |
| 31 | ACAT2 | na |  |  | 12500 | -0.224 | -0.3019 | No |
| 32 | CYP51A1 | na |  |  | 12517 | -0.227 | -0.2981 | No |
| 33 | TM7SF2 | na |  |  | 12627 | -0.237 | -0.2990 | No |
| 34 | FABP5 | na |  |  | 13043 | -0.289 | -0.3148 | No |
| 35 | HMGCR | na |  |  | 13559 | -0.362 | -0.3344 | No |
| 36 | PMVK | na |  |  | 14010 | -0.418 | -0.3494 | Yes |
| 37 | PPARG | na |  |  | 14161 | -0.436 | -0.3484 | Yes |
| 38 | CXCL16 | na |  |  | 14225 | -0.447 | -0.3426 | Yes |
| 39 | FDFT1 | na |  |  | 14374 | -0.474 | -0.3408 | Yes |
| 40 | CLU | na |  |  | 14511 | -0.492 | -0.3379 | Yes |
| 41 | PNRC1 | na |  |  | 14528 | -0.496 | -0.3287 | Yes |
| 42 | LPL | na |  |  | 14944 | -0.553 | -0.3391 | Yes |
| 43 | GSTM2 | na |  |  | 15198 | -0.595 | -0.3403 | Yes |
| 44 | SCD | na |  |  | 15219 | -0.600 | -0.3292 | Yes |
| 45 | LGMN | na |  |  | 15311 | -0.619 | -0.3214 | Yes |
| 46 | GPX8 | na |  |  | 15462 | -0.643 | -0.3162 | Yes |
| 47 | JAG1 | na |  |  | 15843 | -0.713 | -0.3216 | Yes |
| 48 | TP53INP1 | na |  |  | 16090 | -0.768 | -0.3189 | Yes |
| 49 | NFIL3 | na |  |  | 16230 | -0.795 | -0.3100 | Yes |
| 50 | SQLE | na |  |  | 16311 | -0.813 | -0.2977 | Yes |
| 51 | ANTXR2 | na |  |  | 16417 | -0.836 | -0.2862 | Yes |
| 52 | IDI1 | na |  |  | 16426 | -0.838 | -0.2697 | Yes |
| 53 | FAM129A | na |  |  | 16498 | -0.854 | -0.2561 | Yes |
| 54 | HSD17B7 | na |  |  | 16661 | -0.888 | -0.2465 | Yes |
| 55 | STARD4 | na |  |  | 16852 | -0.942 | -0.2373 | Yes |
| 56 | CTNNB1 | na |  |  | 16890 | -0.952 | -0.2199 | Yes |
| 57 | NSDHL | na |  |  | 17265 | -1.072 | -0.2177 | Yes |
| 58 | PLSCR1 | na |  |  | 17416 | -1.120 | -0.2029 | Yes |
| 59 | MAL2 | na |  |  | 17517 | -1.162 | -0.1845 | Yes |
| 60 | PDK3 | na |  |  | 17601 | -1.198 | -0.1646 | Yes |
| 61 | TMEM97 | na |  |  | 17777 | -1.277 | -0.1478 | Yes |
| 62 | ALDOC | na |  |  | 18115 | -1.452 | -0.1359 | Yes |
| 63 | S100A11 | na |  |  | 18222 | -1.511 | -0.1108 | Yes |
| 64 | ERRFI1 | na |  |  | 18341 | -1.595 | -0.0847 | Yes |
| 65 | ANXA5 | na |  |  | 18489 | -1.699 | -0.0579 | Yes |
| 66 | CD9 | na |  |  | 18879 | -2.138 | -0.0348 | Yes |
| 67 | LGALS3 | na |  |  | 19056 | -2.536 | 0.0074 | Yes |
Table: GSEA details [plain text format]

  

Fig 2: HALLMARK\_CHOLESTEROL\_HOMEOSTASIS      
 Blue-Pink O' Gram in the Space of the Analyzed GeneSet

  

Fig 3: HALLMARK\_CHOLESTEROL\_HOMEOSTASIS: Random ES distribution      
 Gene set null distribution of ES for **HALLMARK\_CHOLESTEROL\_HOMEOSTASIS**
